# Evaluation of MR-derived simulated CT-like images and simulated radiographs compared to conventional radiography in patients with shoulder pain: a proof-of-concept study

**DOI:** 10.1186/s12891-022-05076-4

**Published:** 2022-02-05

**Authors:** Georg C. Feuerriegel, Felix K. Kopp, Daniela Pfeiffer, Jonas Pogorzelski, Markus Wurm, Yannik Leonhardt, Christof Boehm, Sophia Kronthaler, Dimitrios C. Karampinos, Jan Neumann, Benedikt J. Schwaiger, Marcus R. Makowski, Klaus Woertler, Alexandra S. Gersing

**Affiliations:** 1 Department of Radiology, Klinikum Rechts der Isar, School of Medicine, Technical University of Munich, Munich, Germany; 2 Department of Orthopaedic Sports Medicine, Klinikum Rechts der Isar, School of Medicine, Technical University of Munich, Munich, Germany; 3 Department of Neuroradiology, Klinikum Rechts der Isar, School of Medicine, Technical University of Munich, Munich, Germany; 4 Department of Neuroradiology, University Hospital of Munich (LMU), Munich, Germany

**Keywords:** Shoulder joint, Magnetic resonance imaging, Radiography, Tomography, X-ray computed, Radiation-free radiography

## Abstract

**Background:**

To evaluate the diagnostic value of MR-derived CT-like images and simulated radiographs compared with conventional radiographs in patients with suspected shoulder pathology.

**Methods:**

3 T MRI of the shoulder including a 3D T1-weighted gradient echo sequence was performed in 25 patients (mean age 52.4 ± 18 years, 13 women) with suspected shoulder pathology. Subsequently a cone-beam forward projection algorithm was used to obtain intensity-inverted CT-like images and simulated radiographs. Two radiologists evaluated the simulated images separately and independently using the conventional radiographs as the standard of reference, including measurements of the image quality, acromiohumeral distance, critical shoulder angle, degenerative joint changes and the acromial type. Additionally, the CT-like MR images were evaluated for glenoid defects, subcortical cysts and calcifications. Agreement between the MR-derived simulated radiographs and conventional radiographs was calculated using Cohen’s Kappa.

**Results:**

Measurements on simulated radiographs and conventional radiographs overall showed a substantial to almost perfect inter- and intra-rater agreement (κ = 0.69–1.00 and κ = 0.65–0.85, respectively). Image quality of the simulated radiographs was rated good to excellent (1.6 ± 0.7 and 1.8 ± 0.6, respectively) by the radiologists. A substantial agreement was found regarding diagnostically relevant features, assessed on Y- and anteroposterior projections (κ = 0.84 and κ = 0.69 for the measurement of the CSA; κ = 0.95 and κ = 0.60 for the measurement of the AHD; κ = 0.77 and κ = 0.77 for grading of the Samilson-Prieto classification; κ = 0.83 and κ = 0.67 for the grading of the Bigliani classification, respectively).

**Conclusion:**

In this proof-of-concept study, clinically relevant features of the shoulder joint were assessed reliably using MR-derived CT-like images and simulated radiographs with an image quality equivalent to conventional radiographs. MR-derived CT-like images and simulated radiographs may provide useful diagnostic information while reducing the amount of radiation exposure.

## Background

Shoulder pain is one of the leading reasons of musculoskeletal consultation in primary care with disorders of the rotator cuff, the glenohumeral and acromioclavicular joint representing the most frequent underlying pathologies [1]. To ensure adequate treatment, basic diagnostic procedures include a thorough medical history, clinical examination as well as conventional radiographs of the shoulder. In clinical routine radiographs are typically acquired in anteroposterior (AP)-projection as well as in Y- projection [2]. Since correct positioning of the shoulder may be challenging, especially in patients suffering from acute shoulder pain, multiple radiographs are sometimes required to achieve acceptable results. Furthermore, in cases with uncertain findings on conventional radiographs or for further assessment of previously detected pathologies, additional computed tomography (CT) is commonly acquired for further assessment of the joint structures. Hence, patients may be exposed to radiation. Therefore, the possibility to assess osseous structures adequately on MR images with the same sensitivity and specificity as with radiation-dependent techniques would be beneficial.

A previous study has demonstrated a feasible approach to acquire radiograph-like images from 3D MR imaging data of the ankle, focusing on the bony surface in order to obtain angle and space measurements [3]. Yet, since only the cortical bone was visualized with this previous technique based on a proton-density weighted sequence, the assessment of the joint and trabecular bone structure was very limited. A more recent study introduced a 3D T1-weighted gradient echo sequence (T1-weighted GRE) which allowed CT- and simulated radiographs to be obtained that were comparable to conventional radiographs regarding their diagnostic value [4]. In order to detect calcifications of the rotator cuff, susceptibility-weighted imaging (SWI) were acquired in patients with calcific tendonitis previously. SWI revealed a a better sensitivity and specificity than standard MR imaging for this specific etiology [5]. Moreover, as a further approach to assess bony structures of the shoulder joint using MR imaging, recent studies have explored the use of Zero Echo Time (ZTE) sequences and Inversion Recovery Ultrashort Echo Time (IR-UTE) sequences. By being able to acquire short T2 values from lamellar bone, UTE as well as ZTE sequences can create a “CT like” contrast, comparable to the contrast of conventional CT scans [6–8]. Additionally, the assessment of bony defects of the shoulder was feasible using UTE and ZTE MR-imaging and comparable to standard CT images [7]. None of these techniques were able to visualize the trabecular bone with a high level of detail, except for the 3D T1-weighted GRE sequence. Moreover, so far, none of these techniques has been used to project radiograph-like images which could be compared to conventional radiographs of the shoulder, which are especially of interest for the assessment of certain pathologies for instance of the acromion and humerus and angle measurements.

Therefore, this proof-of-concept study was designed in order to evaluate the diagnostic value of MR-derived CT-like images and simulated radiographs, based on a 3D T1-weighted GRE sequence, compared to conventional radiographs. In particular the usefulness for the assessment of image quality as well as for the evaluation of semi-quantitative scorings and measurements of the bony geometry of the shoulder joint, which also requires reconstructed projections to be congruent with conventional radiographs, was analyzed.

## Methods

### Patient selection

This study was approved by our institutional review board (Ethics Commission of the Medical Faculty, Technical University of Munich, Germany; Ethics proposal number 258/15 S). Informed consent was obtained from all study participants prior to inclusion. Participants were recruited from February 2019 until March 2020. In total, 25 consecutive patients (mean age 52.4 ± 18 years, 13 women) with shoulder pain diagnosed by one board-certified orthopedic surgeon, were included. All patients had undergone conventional radiography of the shoulder including AP and Y views as part of clinical routine and were scheduled for MR imaging of the shoulder for further evaluation of the ligaments and soft tissue structures of the shoulder joint. In five patients additional CT scans of the shoulder were available. Patients with previous surgical interventions were excluded prior to this study.

### MR imaging

MR imaging was performed using one 3 Tesla MR scanner (Ingenia; Philips Healthcare, Best, The Netherlands) with a dedicated 8-channel shoulder coil (Medical Advances). Standard shoulder imaging protocols were used including triplanar intermediate weighted MR images with fat suppression, a sagittal T2-weighted sequence and a coronal T1-weighted sequence. Additionally, a 3D T1-weighted spoiled gradient echo sequence was acquired in transverse acquisition plane; in-plane resolution, 0.3 mm × 0.3 mm; TE, 2.586 ms; TR, 10 ms; flip angle, 8°; slice thickness, 0.4 mm; standard field of view (FOV) 150x150x80mm^3^. The FOV could be changed slightly depending on the patient size leading to an acquisition time between 4 min 25 s to 5 min 03 s.

### Post-processing

**Fig. 1:**
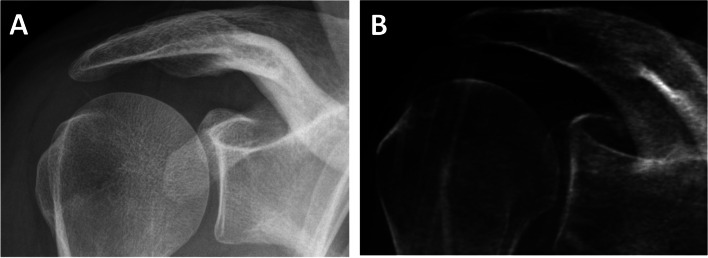
Ideal true AP-projections of the shoulder joint (**A**) on a conventional radiograph and (**B**) on a simulated radiograph. Note the visibility of the glenohumeral joint space and the tangential projection of glenoid fossa in both images

**Fig. 2:**
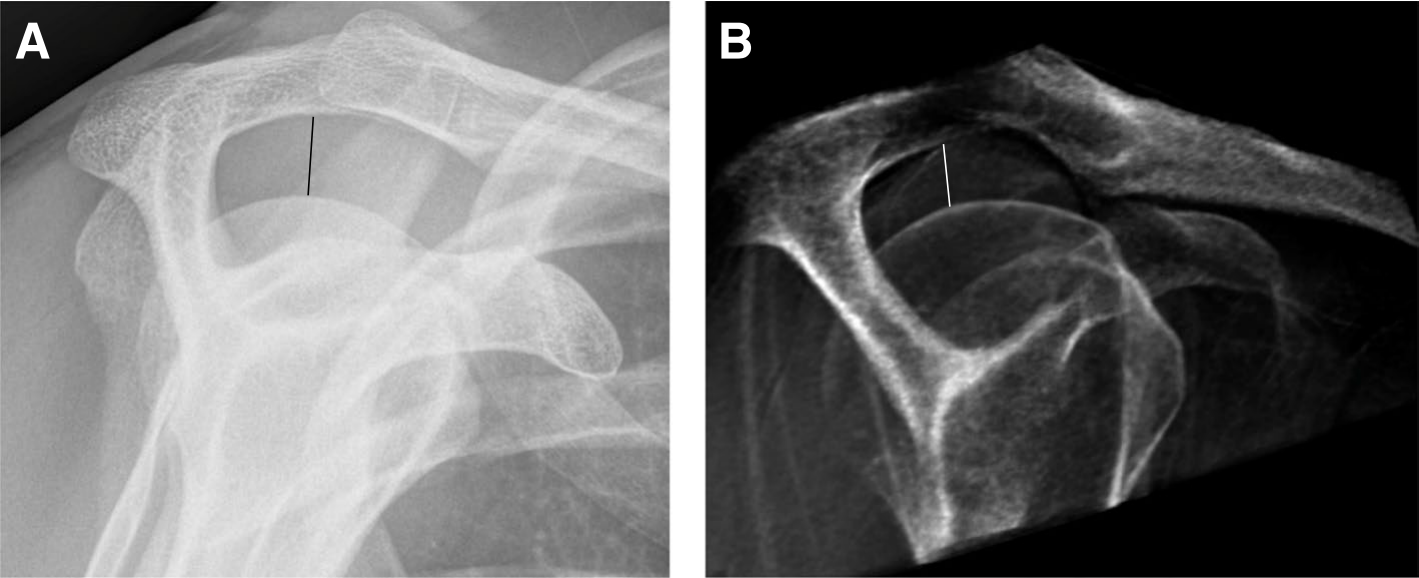
Ideal Y-projections of the shoulder joint (**A**) on a conventional radiograph and (**B**) on a corresponding simulated radiograph. Note the visibility of the acromiohumeral space as well as the position of the humeral head relative to the glenoid. The acromiohumeral distance was measured in both pictures (A = 9 mm in supine position, B = 11 mm in standing position)

A novel software tool built in-house based on MATLAB (MathWorks, Natick, Massachusetts) was used to process the T1-weighted spoiled gradient echo images, as previously reported [4]. On the T1-weighted GRE images the surrounding background was segmented to achieve a binary mask. Subsequently, the intensities of the images were inverted. Further, the contrast of the inverted images was enhanced using an adaptive histogram equalization. In a next step, the 15-th power of the images was computed to further increase the contrast of bone. The processed images were multiplied pointwise with the background mask to avoid disturbing artifacts in the forward projection. In order to generate 2D simulated radiographs, the 3D MR images were processed using a cone-beam forward projection resembling a standard cone-beam CT. In a final step, a diagnostically ideal projection (anterior-posterior-(AP) and Y- projections) of the processed images was chosen for further image analysis (Fig. 1 and Fig. 2).

### Image analysis

Conventional radiographs were used as standard of reference and read beforehand by two board-certified radiologists (B.J.S and A.S.G., both with 9 years of experience in musculoskeletal radiology). After a period of at least 4 weeks, the MR-derived simulated radiographs were analyzed in random order by the same radiologists, which were blinded to clinical information and any other imaging data.

### Semi-quantitative measurements

Overall image quality for each projection (true AP and Y-projection) as well as the visibility of the anatomical landmarks (acromion, glenohumeral joint space, glenoid and acromioclavicular joint) were graded using a four-point Likert scale (1 = excellent visibility/detectability, 2 = good visibility/detectability, 3 = moderate visibility/detectability and 4 = poor visibility/detectability). The overall certainty of imaging features was also graded using a four-point Likert scale (1 = absolute certain, 2 = very certain, 3 = moderate certainty, 4 = not certain due to poor image quality).

**Fig. 3:**
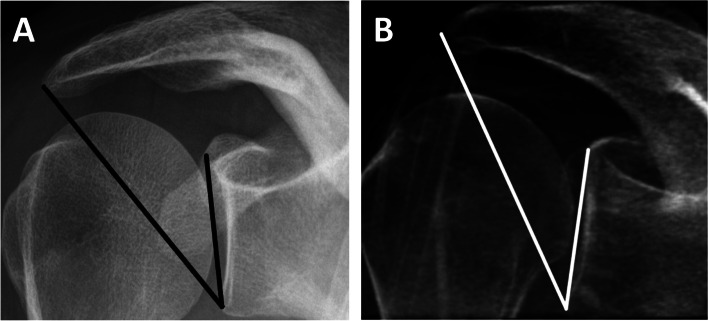
Critical shoulder angle measured (**A**) on a conventional radiograph and (**B**) on a corresponding simulated AP radiograph. A value of 36.8 degrees was measured on both images

For the evaluation of degenerative changes of the glenohumeral joint, the Samilson-Prieto classification was used to grade the stages of osteoarthritis on the basis of the osteophytes sizes located at the inferior humeral head measured in a cranial to caudal direction (Grade I = < 3 mm, mild osteoarthritis, Grade II = 3-6 mm, moderate osteoarthritis, Grad III = ≥7 mm, severe osteoarthritis) [9, 10]. The acromion configuration was assessed according to the Bigliani classification, which categorizes the acromion into three types according to the angle of the lateral notch (type I: flat acromion, type II: curved acromion, type III: hook shaped acromion) [11]. The acromiohumeral distance (AHD) was assessed by measuring the distance from the inferior edge of the acromion to the humeral head in the Y-projection [12, 13]. The critical shoulder angle (CSA; Fig. 3) was measured by drawing a line parallel to the glenoid in an anterior-posterior radiograph and a line through the inferior-lateral edge of the glenoid and the inferior-lateral edge of the acromion.

**Fig. 4:**
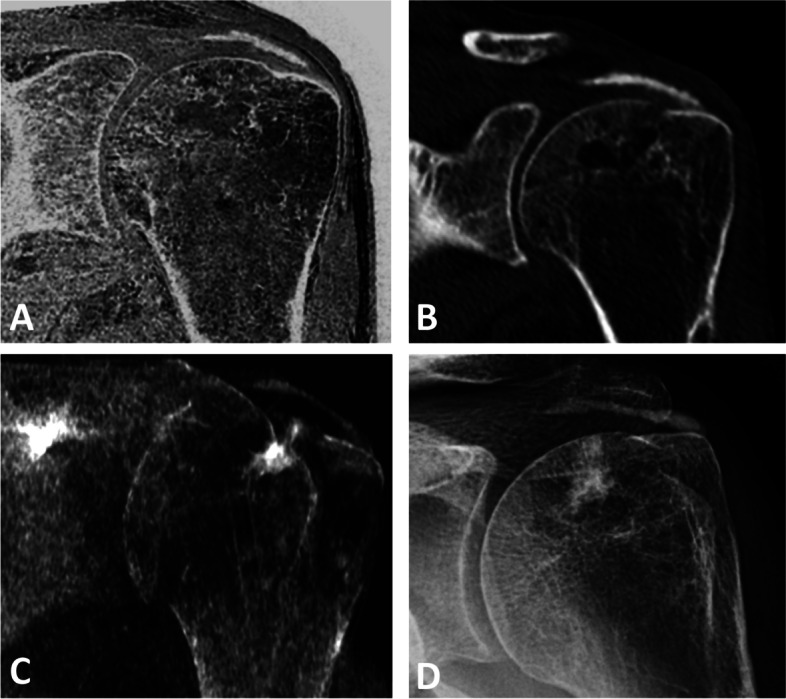
Calcific tendonitis of the left shoulder. **A** Coronal CT-like MR-image, (**B**) conventional CT image, (**C**) MR-derived simulated AP radiograph and (**D**) conventional radiograph correspondingly show linear calcific deposit at the bursal surface of the supraspinatus tendon

**Fig. 5:**
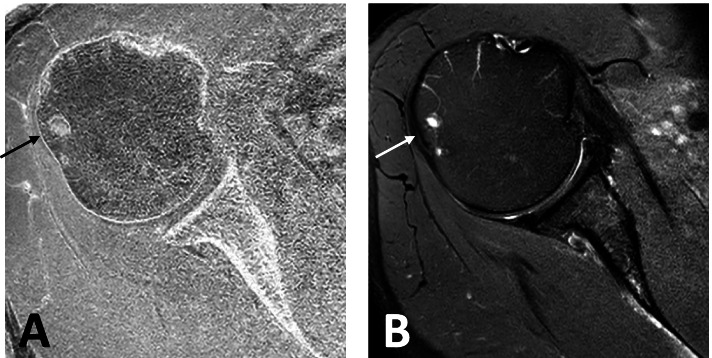
Subcortical cyst of the humeral head. **A** Axial CT-like MR image and (**B**) corresponding intermediate weighted MR- image with fat-suppression showing a small subcortical cyst at the posterior facet of the greater tuberosity. Note the enhanced visibility of trabecular bone structure and cortical delineation of the cysts on CT-like image compared to standard MR image

**Fig. 6:**
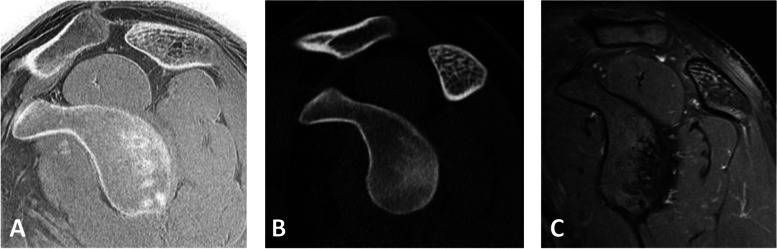
Osseous anatomy of the glenoid. Sagittal (**A**) CT-like MR image, (**B**) conventional CT reformation image, and (**C**) intermediate-weighted MR image show typical pear-shaped morphology of the glenoid. Note the comparable depiction of cortical delineation of the glenoid as well as trabecular bone structure of the acromion on CT- like MR image and conventional CT image

**Fig. 7:**
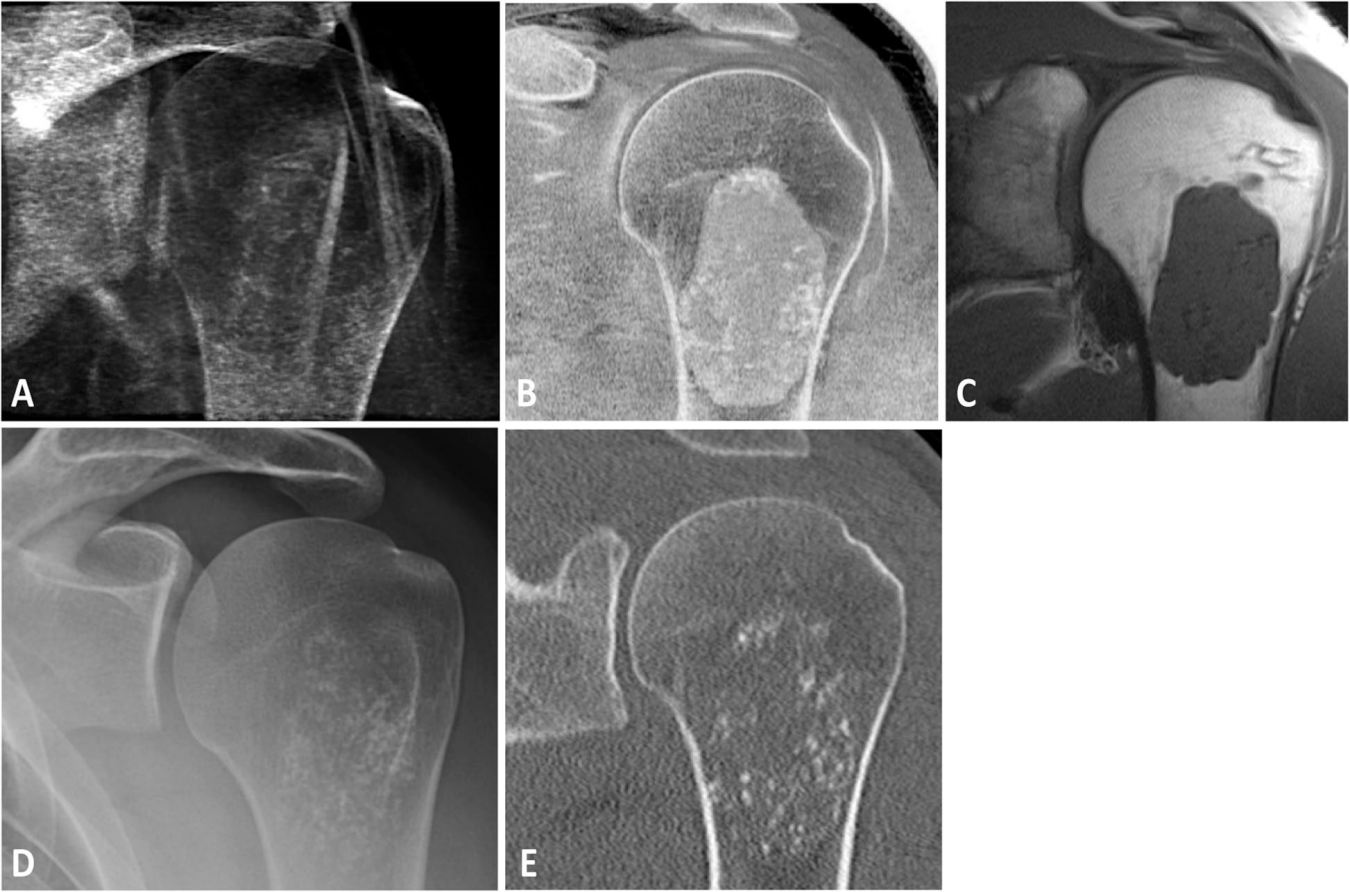
Simulated radiograph (**A**) and corresponding CT-like MR image (**B**) showing chondroid matrix calcifications of an enchondroma at the left proximal humerus in a 28-year-old male patient. (**C**) Standard T1-weighted image showing the enchondroma. Note that the calcifications are not depictable on a routine MR protocol sequence. Additional conventional radiograph (**D**) and CT (**E**) showing the chondroid matrix calcifications

**Fig. 8:**
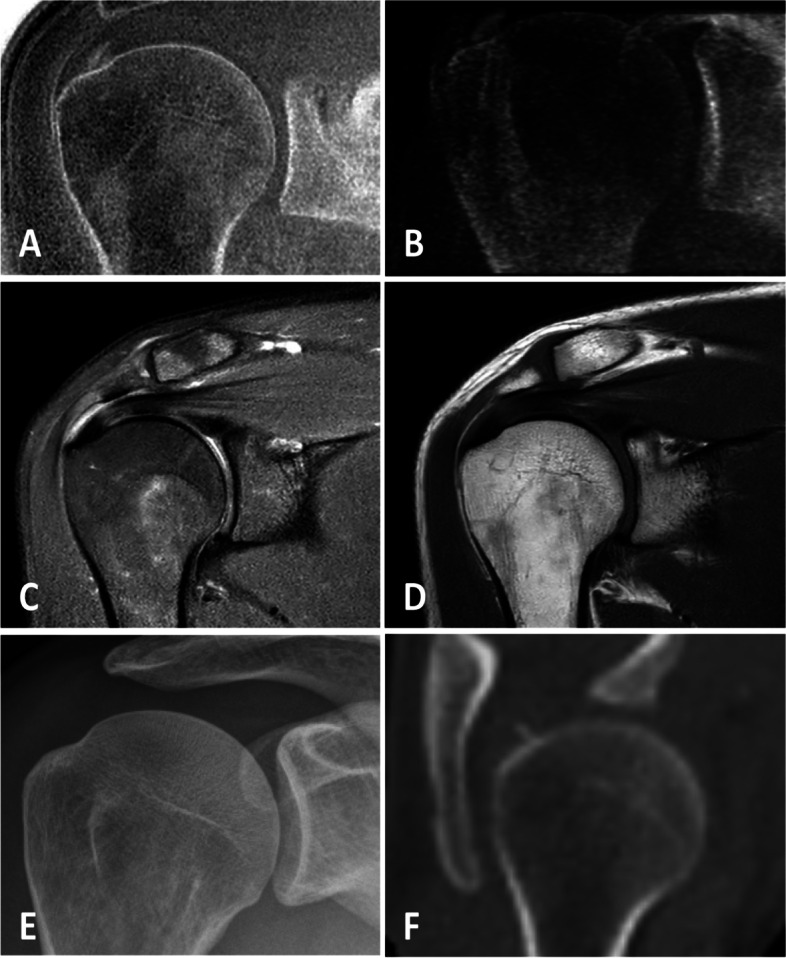
Small calcific deposit can be identified on the CT-like MR images (**A**) and simulated radiographs (**B**) in the supraspinatus tendon. Yet, the calcific deposit was neither clearly circumscribed and depictable on the simultaneously, as part of the clinical routine protocol, acquired intermediate-weighted image with fat saturation (**C**) nor on the T1-weighted image (**D**). Moreover, on the four-weeks preceding conventional radiograph (**E**) the calcification was not depictable, most likely due to the early stage of the calcific tendinitis. An additional CT scan (**F**) that was acquired as part of a chest CT with elevated arms, 3 weeks after the MR images were acquired, showing the small calcified deposition within the supraspinatus tendon

The angle was measured in order to evaluate the humeral coverage of the acromion and the inclination of the glenoid which has been shown to correlate with the risk for rotator cuff tears or glenohumeral osteoarthritis [14, 15]. The CT-like MR images were evaluated by both readers for calcifications of the rotator cuff, osseous glenoid defects, subcortical cysts and tears of the rotator cuff (Fig. 4, 5, 6, 7 and 8). For intra-rater reproducibility, the MR-derived simulated radiographs of the 23 subjects were reassessed after 4 weeks by one radiologist.

### Statistical analysis

The data were analyzed using IBM SPSS Statistics for Windows, version 27.0 (IBM Corp., Armonk, N.Y., USA). All statistical tests were performed two-sided and a level of significance (α) of 0.05 was used for all tests. Paired t-test (for numeric variables), McNemar’s test (for binary categorical variables) and Wilcoxon signed-rank test (for the categorical variables) were used to evaluate differences in gradings between the MR-derived images and the conventional radiographs. Agreement between the MR-derived simulated radiographs and conventional radiographs was calculated using Cohen’s Kappa. The inter-observer and intra-observer reliability of the assessment of the MR-derived simulated radiographs and the assessment of the conventional radiographs were also calculated using Cohen’s Kappa [16]. Agreement was interpreted as poor (0), slight (0.0–0.2), fair (0.21–0.40), moderate (0.41–0.60), substantial (0.61–0.80), and perfect (0.81–1.00) [17].

## Results

Of the 25 patients with shoulder pain included in this study (mean age 52.4 ± 18 years, 13 women), 13 patients (56.5%) showed no significant bony shoulder joint pathologies as depicted on conventional radiographs as the standard of reference. Of the remaining patients, four (33.3%) showed injuries of the rotator cuff (e.g. partial or complete tendon tears), with the supraspinatus tendon being the most often affected (80.0%). Of all patients included into this study, three patients (12.0%) showed abnormalities at the humeral head caused by joint luxation (Hill-Sachs lesions) and two patients (8.0%) showed defects of the labrum and/or the glenoid. Moreover, two patients presented with a calcific tendinitis of the supraspinatus tendon (8.0%) and one patient with an enchondroma of the proximal humerus (4.0%).

### Semi-quantitative image analysis

Diagnostic imaging quality was rated “good” to “excellent” in 22 of the 25 patients (88.0%) included in this study. The agreement of the AP-projections of the MR-derived simulated radiographs and conventional radiographs of these patients was “substantial” to “perfect” for all landmarks for both readers (reader 1, range = 0.78–0.93; reader 2, range = 0.65–0.91; *P* = 0.98; Table 1). The inter-rater agreement for all landmarks of the AP-projections of the MR- derived simulated radiographs compared to the conventional radiographs was “substantial” to “perfect” showing no significant difference between the modalities (MR-derived simulated radiographs, range = 0.71–0.92; *P* = 0.42). The intra-rater agreement of the simulated radiographs, measured by one rater after 4 weeks showed a “substantial” to “perfect” agreement for all landmarks of the AP-projection (MR-derived simulated radiographs, mean 0.77, range [0.76–079]). The image quality of the acromion on the AP-projection of the MR-derived simulated radiographs was rated “good” to “excellent” (reader 1, (mean ± SD) 1.6 ± 0.7; reader 2, (mean ± SD) 1.7 ± 0.6; *P* = 0.42) and showed a high agreement between the two modalities for the AP-projection (κ = 0.92 and κ = 0.91, respectively; Table 2) as well as in the Y-projection (κ = 0.79 and κ = 0.81, respectively).

**Table 1:** Agreement between MR-derived simulated radiographs and conventional radiographs and inter- and intraobserver agreement ^d^

	Agreement between MR-derived simulated and conventional radiographs		Inter- and intraobserver agreement	
	Radiologist 1^a^	Radiologist 2^a^	MR-derived simulated radiographs	
			Interobserver^b^	Intraobserver^c^
**Y-Projection:**				
**Image quality**	1.00 (1.00, 1.00)	0.90 (0.64, 1.00)	0.85 (0.61, 1.00)	0.77 (0.45, 1.00)
**Detectability/visibility of the acromion**	0.79 (0.43, 1.00)	0.81 (0.45, 1.00)	0.81 (0.53, 1.00)	0.72 (0.43, 1.00)
**AHD**	0.95 (0.83, 1.00)	0.60 (0.35, 0.80)	0.90 (0.74, 1.00)	0.85 (0.68, 1.00)
**Acromion configuration**	0.83 (0.60, 1.00)	0.67 (0.38, 0.91)	0.91 (0.73, 1.00)	0.74 (0.44, 1.00)
**True AP-Projection:**				
**Image quality**	0.92 (0.74, 1.00)	0.60 (0.35, 0.91)	0.83 (0.61, 1.00)	0.77 (0.49, 1.00)
**Detectability/visibility of the glenohumeral joint**	0.78 (0.53, 1.00)	0.71 (0.44, 0.92)	0.85 (0.59, 1.00)	0.79 (0.52, 1.00)
**Detectability/visibility of the acromion**	0.92 (0.71, 1.00)	0.91 (0.67, 1.00)	0.92 (0.70, 1.00)	0.77 (0.49, 1.00)
**Detectability/visibility of the glenoid**	0.75 (0.47, 1.00)	0.65 (0.29, 0.91)	0.91 (0.69, 1.00)	0.76 (0.48, 1.00)
**Detectability/visibility of the ac-joint**	0.85 (0.61, 1.00)	0.65 (0.31, 0.91)	0.69 (0.44, 0.91)	0.78 (0.56, 1.00)
**CSA**	0.84 (0.64, 1.00)	0.69 (0.48, 0.86)	0.84 (0,64, 1.00)	0.84 (0.68, 1.00)
**Degeneration by Samilson and Prieto**	0.77 (0.39, 1.00)	0.77 (0.39, 1.00)	1.00 (1.00, 1.00)	0.55 (0.12, 0.89)
**Certainty of imaging features**	0.83 (0.59, 1.00)	1.00 (1.00, 1.00)	0.75 (0.47, 1.00)	0.70 (0.40, 0.92)

^a^Agreement between MR-derived simulated radiographs and conventional radiographs calculated using Cohen κ coefficient

^b^Inter-observer agreement for MR-derived simulated radiograph and conventional radiograph analyses using the Cohen κ coefficient

^c^Intra-observer agreement for MR-derived simulated radiograph and conventional radiograph analyses using the Fleiss κ

*Evaluation using a 4- point Likert -scale

**Table 2:** Mean quality of depiction of radiological landmarks of MR-derived radiographs read by two readers*

	Reader 1		Reader 2	
Y-Projection	**MR-derived radiographs (Mean)**	**Standard deviation**	**MR-derived radiographs**	**Standard deviation**
Image quality	1.6	0.7	1.8	0.6
Detectability/visibility of the acromion	1.9	0.6	2.0	0.5
True AP-Projection				
Image quality	1.6	0.7	1.6	0.6
Detectability/visibility of the glenohumeral joint	1.9	0.8	1.8	0.7
Detectability/visibility of the acromion	1.6	0.7	1.7	0.6
Detectability/visibility of the glenoid	1.7	0.6	1.7	0.5
Detectability/visibility of the ac-joint	1.7	0.7	1.7	0.7
Certainty of the imaging features	1.9	0.6	1.5	0.5

The average AHD (mean ± standard deviation (SD)) was 11.7 ± 1.9 mm and the average CSA was 33.5 ± 3.3°, assessed on conventional radiographs. The agreement between measurements on MR-derived simulated and conventional radiographs for the CSA was “substantial” to “perfect” for both readers (CSA: κ = 0.84 and 0.69, respectively). Regarding degenerative changes of the shoulder joint (Samilson- Prieto classification), 74% of the shoulders were assessed as grade I, 26% as grade II and 0% as grade III by both readers. Moreover, 44% of the patients showed a type 1 acromion configuration (type I, *n* = 11). Again, the agreement between MR-derived simulated and conventional radiographs for the two scores was “substantial” to “perfect” (Samilson score: κ = 0.77 and 0.77; acromion configuration: κ = 0.83 and κ = 0.67, respectively).

The inter-rater agreement for the measurements of the AHD and CSA was “substantial” to “perfect” (AHD, κ =0.90; CSA, κ = 0.84). Indicating a high reproducibility, the intra-rater agreement of the measurements of the AHD and CSA on the simulated radiographs was rated “perfect” (AHD, κ =0.85 and CSA, κ = 0.84,).

Additionally, in one patient an osseous glenoid defect (4.0%) was found after shoulder joint dislocation and it was detected on both modalities by both readers (100% for both; Fig. 5, Table 3). In two further patients calcific tendinitis of the supraspinatus tendon was detected (Figs. 4 and 8). It is important to note that both readers evaluated the visibility of those features on the CT images as well as CT-like MR images equally good (reader 1, (mean ± SD) 1.3 ± 0.6; reader 2, 1.7 ± 0.6; *P* = 0.42). In two further patients a subcortical cyst was detected by both readers on the MR-derived CT-like images due to the fact that fluid content is depicted as hyperintensity on the inverted images, whereas on the corresponding CT-scan the readers could not easily differentiate between an osseous defect and a subcortical cyst (Fig. 5).

**Table 3:** Visualization of osseous features both seen on the CT-like T1 GRE MR sequences and conventional CT scans^a^

No. of cases	**Features visualized**
2	Subcortical cyst of the humeral
2	Calcifications of the rotator cuff
1	Glenoid defects (bony Bankart)
1	Enchondroma of the proximal humerus

^a^Osseous features were detected in 4 of the 5 cases where additional CT-scans were available

## Discussion

This study was able to demonstrate that the agreement of angle and distance measurements as well as morphological gradings between conventional radiographs and simulated radiographs, obtained from a MR-based 3D T1-weighted GRE sequence, are excellent, suggesting that the MR-derived simulated radiographs may be a feasible technique for the evaluation and assessment of osseous shoulder pathologies. Moreover, for changes which cannot be detected on projectional radiography, MR-derived CT-like images provided additional information on the subcortical and trabecular bone.

Conventional radiography and CT are commonly used for the evaluation of osseous structures in clinical routine. CT images may be acquired in patients with shoulder pain due to its higher sensitivity regarding the detection of osseous pathologies, especially in regions in which radiography is limited due to superimposition, e.g. for the detection of a lesion of the glenoid or for the assessment of the presence and exact localization of calcifications [18]. However, a previous MR-based study comparing MR and CT images of the glenoid has demonstrated that MR images of the glenoid were almost as accurate as CT-based images when assessing the glenoid width [19]. This underlines the possible use of MR-based techniques for radiation-free imaging of bone. While MR-derived CT-like images provided additional information on the osseous structures of the glenohumeral joint in our study, the creation of simulated radiographs may be an important technique in order to acquire radiation-free radiograph-like images of the glenohumeral joint, especially in young patients if no radiograph or CT has been performed previously. Semi-quantitative shoulder joint assessment scores and angle measurements performed using simulated radiograph-like images may be useful for the evaluation of the osseous structures of the shoulder for therapy planning and monitoring [20]. This MR-based technique may lead to less use of conventional radiographs and CT scans in the future, since the diagnostic information could be acquired in one examination using MR imaging. This would be particularly useful for children and young adults, as this examination would imply no use of radiation.

To the best of our knowledge, this is the first study to assess MR-based simulated radiographs of the shoulder. Nordeck et al. assessed ankle joints using MR-based simulated radiographic bone models based on proton-density weighted images [3]. Angle measurements were performed on these images after cortical bone had been segmented in 3D MR datasets [4]. In contrast to this previous study, we were able to maintain the information on the osseous structure, especially of the trabecular bone, without segmentation of cortical bone, which is especially relevant for the detection of lesions within the bone, e.g. cystic changes or tumorous lesions. Due to the high resolution of the T1-weighted GRE sequence, trabecular bone is visualized with a similar quality compared to the quality of CT images, which is a major benefit of this sequence compared to the other current bone imaging MR-based techniques [5, 6, 21]. Moreover, especially when assessing CT-like images, the image dataset only had to be inverted with no additional post processing, which is a major advantage to the previously reported studies on MR-imaging of bone structures with time consuming post processing algorithms [6]. In this study, the post-processing in order to generate simulated radiographs was performed by virtually simulating the tube-detector distance of conventional radiographs, the detector size and the x-ray cone beam angle. Therefore, no image information on cortical or trabecular bone was lost due to image post-processing procedures, as described previously [3]. A previous study has shown that the evaluation of the destruction patterns and periosteal reactions in patients with benign or malignant bone tumors is feasible using MR- derived simulated radiographs and CT-like images based on T1-weighted GRE imaging [4]. CT-like images derived from the T1-weighted GRE sequences provide information on both cortical as we well as trabecular bone, which allows the visualization and detection of pathologies of the trabecular bone and subcortical structures, such as subcortical cysts and fractures, which is not possible after segmenting the cortical bone, as described in the previous study [3].

A previous study identified calcifications in patients with calcific tendonitis using SWI, acquired at a 1.5 Tesla MRI. The in-plane resolution and section thickness, which are crucial for the detailed assessment of osseous structures, were limited in this previous study compared to our study [5]. With the T1-weighted GRE sequence used in this study we were able to achieve a 0.4 × 0.4 × 0.4 mm resolution, which was very useful for the detailed depiction of trabecular bone structures. Several studies using ZTE and IR UTE were performed at the shoulder and the hip in order to create CT-like images [6–8, 22]. To achieve a comparable resolution with UTE and ZTE sequences it would require significantly longer acquisition times. The morphology of these CT-like images was comparable to standard CT images in all studies. Yet, assessment of the trabecular bone structure was compromised in UTE and ZTE techniques compared to the T1-weighted GRE sequence, due to the lower resolution und larger voxel size. Moreover, the T1-weighted GRE sequence can be acquired as 3D sequence, and due to the isotropic voxel, reconstructions could be obtained in every plane needed. Additionally, further time-consuming post-processing was needed in the UTE and ZTE studies in addition to the acquisition of the sequence.

Our study has limitations. Tissues with short T2-relaxations times are projected hyperintense in the inverted T1-weighted GRE sequence, including bone as well as e.g. ligaments or menisci. However, these structures do not entirely obstruct the depiction of bony structures and abnormalities and are mostly subtracted in the post-processing of the simulated radiographs. Another limitation is the need for a 3 T MRI which might not be available at every hospital. Also, image acquisition using a 1.5 T MRI has not been tested yet and needs to be looked at in the future. Furthermore, 3 T MRI is an expensive examination compared to conventional radiographs and might not be suitable for every institution. This topic needs to be evaluated in future as the cost -effectiveness analyses. For the post-processing and the acquisition of the MR-derived simulated radiographs we used automated computerized algorithms on dedicated workstations which are up to now not part of the regular clinical set up. Additionally, the post processing of the MR images took about 5 min per examination. The duration of this process needs to be shortened in the future to make it suitable for clinical routine. Furthermore, it needs to be noted, that this is a proof-of-concept study, with a small sample size. Future studies in larger study cohorts with specific pathologies are needed for further evaluation of this technique.

## Conclusion

Reconstructions of simulated radiographs of the shoulder joint based on the T1-weighted GRE MR-derived CT-like images were feasible using a novel cone-beam forward projection algorithm. Additionally, the assessment of clinically relevant features of the shoulder joint using MR-derived CT-like images and simulated radiographs revealed a quality equivalent to that of conventional radiographs. CT-like images and simulated radiographs therefore may be a useful radiation-free technique, which may provide additional diagnostic information on osseous structures for the detailed assessment of the shoulder joint, which is especially relevant in younger patients.

## Data Availability

The data that support the findings of this study are not openly available due to reasons of sensitivity of human data and are available from the corresponding author upon reasonable request.

## Abbreviations

MRI: Magnetic resonance imaging
CT: Computer tomography
AP: Anterior posterior
CSA: Critical shoulder angle
AHD: Acromiohumeral distance
SWI: Susceptibility-weighted images
T1 GRE: T1-weighted gradient-echo sequence
UTE: Ultrashort echo time
ZTE: Zero Echo Time
TE: Echo time
TR: Repetition time
FOV: Field of view

## References

[1] Mitchell C (2005). Shoulder pain: diagnosis and management in primary care. BMJ.

[2] Nazarian LN (2013). Imaging algorithms for evaluating suspected rotator cuff disease: Society of Radiologists in ultrasound consensus conference statement. Radiology.

[3] Nordeck SM (2017). Simulated radiographic bone and joint modeling from 3D ankle MRI: feasibility and comparison with radiographs and 2D MRI. Skelet Radiol.

[4] Gersing AS (2019). Evaluation of MR-derived CT-like images and simulated radiographs compared to conventional radiography in patients with benign and malignant bone tumors. Eur Radiol.

[5] Norenberg D (2016). Diagnosis of calcific tendonitis of the rotator cuff by using susceptibility-weighted MR imaging. Radiology.

[6] Breighner RE (2018). Technical developments: zero Echo time imaging of the shoulder: enhanced osseous detail by using MR imaging. Radiology.

[7] Ma YJ (2018). Feasibility of using an inversion-recovery ultrashort echo time (UTE) sequence for quantification of glenoid bone loss. Skelet Radiol.

[8] de Mello RAF (2020). Three-dimensional zero Echo time magnetic resonance imaging versus 3-dimensional computed tomography for glenoid bone assessment. Arthroscopy.

[9] Samilson RL, Prieto V (1983). Dislocation arthropathy of the shoulder. J Bone Joint Surg Am.

[10] Habermeyer P (2017). Classification of humeral head pathomorphology in primary osteoarthritis: a radiographic and in vivo photographic analysis. J Shoulder Elb Surg.

[11] Pandey V, Jaap Willems W (2015). Rotator cuff tear: a detailed update. Asia Pac J Sports Med Arthrosc Rehabil Technol.

[12] Brolin TJ, Updegrove GF, Horneff JG (2017). Classifications in brief: Hamada classification of massive rotator cuff tears. Clin Orthop Relat Res.

[13] Hamada K (1990). Roentgenographic findings in massive rotator cuff tears. A long-term observation. Clin Orthop Relat Res.

[14] Viehofer AF (2016). A larger critical shoulder angle requires more rotator cuff activity to preserve joint stability. J Orthop Res.

[15] Cherchi L (2016). Critical shoulder angle: measurement reproducibility and correlation with rotator cuff tendon tears. Orthop Traumatol Surg Res.

[16] Fleiss JL, Cohen J (1973). The equivalence of weighted kappa and the Intraclass correlation coefficient as measures of reliability. Educ Psychol Meas.

[17] Fleiss JL (1971). Measuring nominal scale agreement among many raters. Psychol Bull.

[18] Farin PU (1996). Consistency of rotator-cuff calcifications. Observations on plain radiography, sonography, computed tomography, and at needle treatment. Investig Radiol.

[19] Lee RK (2013). Glenoid bone loss: assessment with MR imaging. Radiology.

[20] Stamiris D (2020). Critical shoulder angle is intrinsically associated with the development of degenerative shoulder diseases: a systematic review. Orthop Rev.

[21] Schwaiger BJ, Schneider C, Kronthaler S, Gassert FT, Böhm C, Pfeiffer D, Baum T, Kirschke JS, Karampinos DC, Makowski MR, Woertler K, Wurm M, Gersing AS. CT-like images based on T1 spoiled gradient-echo and ultrashort echo time MRI sequences for the assessment of vertebral fractures and degenerative bone changes of the spine. Eur Radiol. 2021;31(7):4680-9. 10.1007/s00330-020-07597-9. Epub 2021 Jan 14. 10.1007/s00330-020-07597-9 PMC8213670 33443599

[22] Breighner RE, et al. Evaluation of osseous morphology of the hip using zero Echo time magnetic resonance imaging. Am J Sports Med. 2019;47(14):3460–8. 10.1177/0363546519878170 31633993

